# Defining score interpretation thresholds for clinical outcome assessments: a review of terminology and reporting recommendations

**DOI:** 10.1186/s41687-025-00966-2

**Published:** 2025-11-26

**Authors:** E. Flood, N. Clarke, B. L. King-Kallimanis, J. Musoro, S. Eremenco, C. L. Ward, J. C. Cappelleri, S. Nolte

**Affiliations:** 1 Patient Centered Science, AstraZeneca, Gaithersburg, MD USA; 2 LUNGevity Foundation Institution, Bethesda, MD USA; 3 European Organisation for Research and Treatment of Cancer (EORTC), Brussels, Belgium; 4 Critical Path Institute, Tucson, AZ USA; 5 Otsuka Pharmaceutical Development & Commercialization Inc., Rockville, MD USA; 6 Statistical Research and Data Science Center, Pfizer Inc., New York, NY USA; 7 Person-Centred Research, Eastern Health Clinical School, Monash University, Melbourne, Victoria, Australia

**Keywords:** Clinical outcome assessment, Patient-reported outcomes, Self-Report, Minimal important difference, MID, Minimal clinically important difference, MCID, Meaningful change

## Abstract

**Purpose:**

The concept of ‘score interpretation threshold’ for understanding score differences of clinical outcome assessments (COAs) and terminology around this topic have evolved over several decades. Yet, considerable confusion regarding terminology remains, leading to potentially erroneous interpretation of COA results. This article sought to provide an updated overview of terminology and an assessment of trends to explore opportunities for harmonizing the field.

**Methods:**

A targeted literature review was conducted for review articles published 2016-September 2024 discussing terminology related to COA score interpretation thresholds, followed by a review of guidance by regulatory and reimbursement/payer stakeholders for specific terminology in this context. A targeted review of original research articles that were aimed at deriving interpretation thresholds was undertaken, spanning a five-year period (2016- Apr 2021) to explore potential trends regarding use of terms, acronyms, and definitions.

**Results:**

As expected, vast heterogeneity in terminology and definitions was observed across review articles and regulatory/reimbursement/payer guidelines. Across 318 original research articles, 39 different terms were identified, with ‘minimal clinically important difference’ (MCID) most frequently used (mentioned in 163 articles), which was more than twice as often as the next term (‘minimal important difference’; MID), mentioned in 76 articles, followed by ‘minimal important change’ (MIC), mentioned in 54 articles. Articles also showed great variation in how thresholds were defined, derived, and applied. Frequently, authors failed to provide sufficient details on methods and application, making it difficult to interpret derived thresholds.

**Conclusions:**

COA score interpretation threshold terminology is far from harmonized. Evidence is insufficient to derive specific recommendations on which terms to use. Instead, we present minimum reporting standards for defining thresholds to ensure that they are comprehensible and reproducible, regardless of the specific terms and acronyms used.

## Background

Clinical or other health interventions are typically aimed at improving symptoms or other aspects of a person’s health-related quality of life (HRQoL), such as physical, emotional, or social functioning. In situations where improvement or cure cannot be expected, maintenance of symptom or functioning levels – in other words avoidance of deterioration – may be an alternative treatment aim. In order to interpret if an intervention is providing benefit, whether that be improvement or maintenance, it is critical to know what amount of change over time in Clinical Outcome Assessment (COA) score at the individual or single group mean level – or what difference in mean change score between groups – can be considered ‘meaningful’ [1, 2]. In this context, there are several scenarios where the definition of a threshold is important. In situations without a direct comparator, change may refer to change within an individual (i.e., within-person change over time) or change within a (single) group of patients (i.e., within-group change over time). In contrast, in situations with direct comparators, the interpretation of score difference may refer to the magnitude of difference between two or more groups, for example, in randomized controlled trials (i.e., difference in mean change between groups) [3]. Finally, it may also be of interest to compare an individual or a group of patients to reference values based on other patients’ HRQoL [4] or to norm data obtained from the general population [5], using the example of reference values published for the cancer-specific HRQoL questionnaire QLQ-C30 of the European Organisation for Research and Treatment of Cancer (EORTC) [6]. The latter scenarios are further examples of where it is crucial to understand what magnitude of difference between an individual or a patient group can be considered a ‘meaningful’ difference compared to a reference population.

To understand the development of the field of COA score interpretation thresholds, we took a brief look at the evolution of terminology. The purpose is to provide a brief overview of terms and abbreviations that we believe have received the most attention in this area over the last decades, without claiming completeness.

As early as the mid- to late 1980s, the first studies were published on the interpretation of change or difference as measured by self-reported psychological or quality of life questionnaires. By deriving cut-off points, patients could be categorized in terms of having/not having experienced ‘meaningful’ change. In 1987, Guyatt and colleagues [1] first introduced the term ‘Minimal Clinically Important Difference’ (but not MCID as an abbreviation). The term was introduced in the context of responsiveness, i.e., one of the key attributes to evaluate self-report measures. Two years later, Jaeschke, Singer and Guyatt (1989) published their seminal paper on MCID, introducing the abbreviation ‘MCID’ and providing the first formal – and frequently cited – definition of MCID as: “(…) the smallest difference in score in the domain of interest which patients perceive as beneficial and which would mandate, in the absence of troublesome side effects and excessive cost, a change in the patient’s management” [2]. Shortly after, Jaeschke et al. (1991) [7] introduced the concept of ‘Minimal Important Difference’ (MID) that – in its definition – remained fairly close to that of MCID; however, they emphasized that ‘clinically’, i.e., the notion of ‘clinician input’ should be avoided, as an MID should be based on the patient’s experience.

Substantial further research activity then occurred in the first decade of this century, with terms like ‘Minimally Clinically Important Change’ (MCIC), introduced as a counterpart to MCID [8, 9], and ‘Minimal Clinically Important Improvement’ (MCII) for use in clinical trials, with the argument that trials always aim for improvement (rather than worsening) and the magnitude of thresholds may differ between improvement versus worsening [10]. A closely related term to the MCID, the ‘Minimally Important Change’ (MIC), was introduced shortly after the introduction of MCIC, thereby specifying the need for it to be derived using anchor-based methods, as opposed to statistically derived (i.e., distribution-dependent) thresholds [11]. In parallel, Tubach et al. [12] proposed a contextual distinction between MCID and ‘Patient Acceptable Symptom State’ (PASS), with the latter defined as the value beyond which patients consider themselves ‘well’. Hence, PASS addresses the concept of well-being or remission (i.e., ‘feeling good’) as opposed to – and complementary to – the concept of MCID (i.e., ‘feeling better’).

Yet another concept introduced around that time was the ‘Clinically Important Difference’ (CID), with group-level differences defined as ‘clinically important’ but not necessarily ‘minimal’ [13]. Cappelleri et al. (2013) [14] further iterated that, by using aforementioned anchor-based methods, with the anchor being a global assessment item, the smallest clinically measurable difference on the target COA is defined as a pair of adjacent categories on the global assessment item. Therefore, the anchor is restricted by its scale and the identified ‘important difference’ may not equate to a ‘minimally’ important difference but would be a ‘clinically important’ one [15, 16]. A few years later, ‘Substantial Clinical Benefit’ (SCB) was introduced as an alternative concept to assess treatment effectiveness. The authors argued that the MCID was a floor value and should not be a true treatment goal; hence, the SCB was defined as a threshold for patients to report they are ‘much better’ [17, 18]. In the context of individual-level change, the concept of ‘Clinically Important Responder’ (CIR) was introduced in 2015 [19, 20]. As opposed to the group-level CID threshold, the CIR was defined as “(…) the amount of change a patient would need to report to indicate that a relevant treatment benefit has been experienced.” Categorizing patients as responders versus non-responders (akin to the ‘responder definition’ as introduced in the FDA 2009 Guidance [21]) allows for the evaluation of differences between treatment and comparator groups [20].

Apart from various terms and acronyms introduced over the last several decades, some key initiatives have occurred simultaneously aimed at improving the harmonization and standardization of COA score interpretation thresholds. First, the Clinical Significance Consensus Meeting Group published a series of papers on this topic. It is beyond the scope of the present introduction to describe the many papers included in this series, but the interested reader may refer to our references [22–37]. Second, early in 2015, the Consensus Panel for Outcomes Measurement and Psychometrics: Advancing the Scientific Standards (COMPASS) was initiated by Clinical Outcomes Solutions (COS), also a sponsor of COMPASS together with the Critical Path Institute (C-Path) and The University of Arizona. The Panel’s goal was to tackle psychometric and methodological issues facing the COA field, with one key topic being consensus on ‘meaningful change’. Third, is the electronic database PROMID, an inventory of anchor-based MID estimates for patient-reported outcome (PRO) measures (http://www.promid.org), which is an initiative by Prof Guyatt and team at McMaster University, Hamilton, Canada [38]. The inventory is a resource to aid the interpretation of PRO scores, including a credibility instrument to assess the quality of published MIDs [39]. Finally, the Setting International Standards in Analyzing Patient-Reported Outcomes and Quality of Life Endpoints in Cancer Clinical Trials-Innovative Medicines Initiative (SISAQOL-IMI) Consortium was launched in 2021 with the goal to improve design, statistical analysis, and reporting of PRO data in cancer clinical trials, with one sub-objective focusing on clinically meaningful change, including terminology [40].

Despite the many terms denoting ‘COA score interpretation thresholds’ as well as initiatives to progress the field, there remains a large amount of inconsistency – and oftentimes frustration – around the topic. Therefore, the Study Endpoints, Clinical Research, and Statistics & Data Science Communities of the Drug Information Association (DIA) launched four cross-community Meaningful Change Working Groups (WGs) in 2021. The objectives of these WGs were to undertake a systematic approach to the topic of COA score interpretation thresholds. This included a review of the history and evolution of terms, definitions and acronyms used by different COA stakeholders (WG 1, the present article), contextualization of thresholds regarding different stakeholder perspectives (WG 2) [41], exploration of methods used to derive thresholds (WG 3), and the definition of standards for evaluating thresholds with digital health technology (WG 4) [42]. Apart from the stated review of terms, the objective of WG1 was to uncover potential trends towards common usage of language (i.e., terms, acronyms, definitions) across different stakeholder groups with the ultimate aim to contribute to the harmonization and streamlining of terminology. Lastly, we present minimum reporting standards for defining score interpretation thresholds for use in future publications of COA score interpretation thresholds.

## Methods

In order to provide a comprehensive overview of terminology and definitions around COA score interpretation thresholds and explore the status quo regarding use of terminology across stakeholders, several data sources were explored with a focus on understanding:

1. terms and definitions used in methodology review papers;
2. use in guidance documents by regulators/health technology assessment (HTA) agencies/payers; and
3. real-world use of terms in research articles aimed at deriving COA score interpretation thresholds.

A targeted literature review was initially conducted in July 2021, covering literature published during the preceding 5-year period (2016 – July 2021). Using MEDLINE and Embase, this review was to support the activities of all four DIA WGs. The search was restricted to humans and English-language publications. The terms used are provided in the Supplemental Material. An update of the literature search, limited to methodological reviews, was then conducted in September 2024 by WG1 using the same search strategy as the original targeted review described above. Titles and abstracts were reviewed for relevance to address the objectives of each WG. Abstracts meeting one of the following criteria were tagged for WG1:

*Terms and definitions used in methodology review papers:*

- Provided a history of score interpretation threshold terminology and definitions;
- Provided commentary on terms related to the concept of score interpretation thresholds; or
- Provided recommendations for the use of score interpretation threshold terminology.

*Real-world use of terms in threshold derivation articles:*

- Objective of the study was to derive score interpretation thresholds for COA(s);
- Studies applying existing score interpretation thresholds to interpret COA data were excluded.

All abstract screening was conducted by members of the DIA Meaningful Change Working Group. A single screening was conducted, and those tagged for WG1 were reviewed by a second member of the DIA working group to determine relevance for full paper review.

**Fig. 1:**
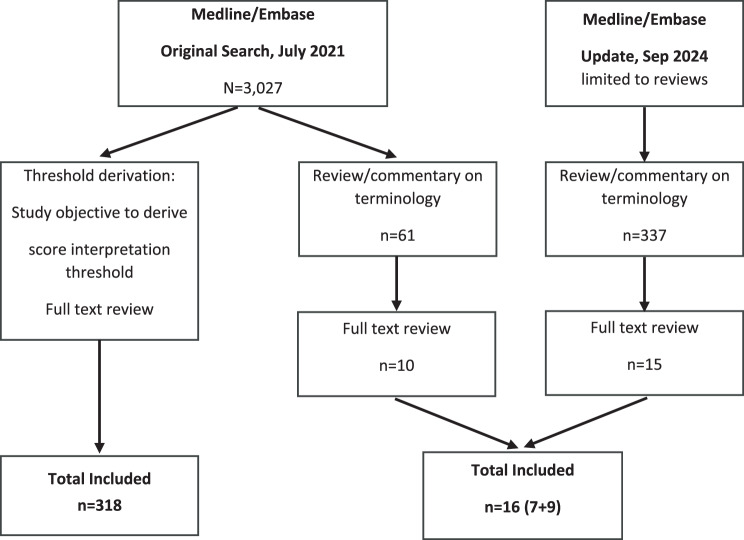
Prisma flow chart for the targeted literature review

A PRISMA flow chart of the targeted literature review is provided in Fig. 1.

### Review of terms and definitions used in methodology review papers

Reviews from the original and updated search meeting the eligibility criteria were selected and information tabulated by WG1, with review of extracted data by a second WG1 member for quality assurance. Data extracted included the specific terms used and corresponding definitions, as well as direct quotes of author commentary on terminology.

### Review of guidelines from regulatory and HTA/payer organizations

Regulatory, HTA and Payer organizations were selected by the WG to represent key drug approval processes globally. An internet search of the selected regulatory body and HTA/payer organization websites was conducted in 2024 to identify any published guidance (excluding indication-specific guidance) from these stakeholders utilizing terms to describe COA score interpretation thresholds. For regulatory authorities, we reviewed guidance documents published by the US (Food and Drug Administration, FDA) and Europe (European Medicines Agency, EMA). Guidance published by China (Center for Drug Evaluation, CDE) was also identified but was not available in English and therefore excluded. For HTA/Payers, we reviewed published guidance documents from organizations in Australia, Canada, France, Germany, Japan, Scotland, Sweden, UK, US and the European Member State Coordination Group on Health Technology Assessment (HTA CG), Directorate-General for Health and Food Safety, formerly European network for Health Technology Assessment (EUnetHTA).

### Review of threshold derivation papers

The initial targeted literature search (July 2021) identified papers that described original studies aimed at deriving COA score interpretation thresholds. After removing duplicates and papers that did not describe the derivation of thresholds, relevant articles were tabulated. Data extraction and quality control (QC) were conducted by IQVIA research staff with expertise in PRO/COA research. QC was conducted on 10% of articles in two steps: 1) the initial 5% of articles extracted were reviewed as they were completed to ensure accuracy and consistency across the extraction team; 2) 5% of remaining articles for QC were selected randomly. Data extracted related to WG1 objectives included the specific score interpretation threshold terms, acronyms, definitions and references for definitions used. Results were summarized in total and by therapeutic area.

## Results

### Overview of published methodology review articles

The initial targeted literature search conducted for the period 2016 to July 2021 yielded 3,027 articles. Of these, 61 articles were identified as relevant methodology review articles for WG1 objectives. After abstract review, 10 articles were identified for full article review, of which seven were deemed relevant and included in the review. The updated targeted literature search (September 2024), limited to review articles, yielded an additional 337 articles. After abstract review, 15 articles were identified for full text review, and of these, nine were included in the review. Hence, a final number of 16 review articles that defined and provided a review and/or commentary on terminology used to describe COA score interpretation thresholds were tabulated (Table 1).

**Table 1:** Overview of terms and comments in methodological review articles, published between 2016 and 2024

Reference	Objective	Terms mentioned	Terms defined and definitions	Other comments on terminology
Coon and Cappelleri 2016 [20]	To provide an overview of the presentations at the 6^th^ Annual PRO Consortium Workshop (April 2015) that focused on the interpretation of change in PRO scores as well as other COAs	• Minimum clinically important difference (MCID) • Minimum important difference (MID) • Clinically important difference (CID) • Clinically important responder (CIR)	• *MCID*: “The smallest difference in score in the domain of interest which patients perceive as beneficial and which would mandate, in the absence of troublesome side effects and excessive cost, a change in the patient’s management.’’ *(quoting Jaeschke et al., 1989* [2]) • *MID*: “(…) differs from MCID in that it may be established without a clinical judgment” *(referencing Juniper et al., 1994* [43]) • *CID*: “(…) difference in scores between 2 treatment groups that can be considered clinically relevant” • *CIR*: “(…) the amount of change a patient would have to report to indicate that a relevant treatment benefit has been experienced”	• “(…) MCID was introduced and evaluated in the context of interpreting the meaning of group-level change scores in a study (ie, group mean change), although the authors noted that the results can be used ‘in interpreting questionnaire scores, both in individuals and in groups of patients participating in controlled trials’. Thus, from its outset, MCID was used interchangeably to interpret group-level as well as individual-level change.” • “(…) the *minimum important difference* (MID, which differs from MCID in that it may be established without a clinical judgment [*quoting the FDA draft PRO Guidance 2006* [44]]) was to be used for interpreting group-level mean differences, while the responder definition was to be used for characterizing individuals as treatment responders. In fact, the draft PRO Guidance touched on the difference in magnitude between these 2 thresholds, suggesting that meaningful change for defining individual responders would be a higher or larger threshold than the MID threshold, which corresponds to the smallest meaningful group-level effect.” • “Although the term ‘MID’ was excluded in the final PRO Guidance, the field continues to employ this concept, in particular for conducting power calculations when planning clinical trials. However, despite the draft PRO Guidance clearly distinguishing between the importance of change within-patient and between-group, the term ‘MID’ is sometimes erroneously used for these 2 concepts interchangeably. Given the long history, inconsistent use, and vagueness of ‘minimal’, some PRO experts have suggested that we eliminate the terms ‘MID’’’ and ‘MCID’ from our vocabulary. In particular, a minimal amount of change may be noticeable to patients, but it does not necessarily mean that it is important to patients.” • “These terms ‘CID’ and ‘CIR’ have an intuitive rationale in that ‘difference’ matches up with mean differences between groups and ‘responder’ matches up with changes within individuals. Further, these terms allow for us to retire the term ‘MID’ and focus on the clinical interpretation of the threshold rather than just the magnitude of the threshold.”
Collins 2019 [45]	To present a schema for understanding measurement of clinical significance and highlight reasons why misuse and misinterpretation have occurred	• Minimal clinically important difference (MCID) • Clinically important difference • Minimally important change • Minimal important difference • Minimal detectable change	• *MCID*: “(…) the smallest change in an outcome measure that results in an important change as identified by the patient or which represents meaningful improvement as judged by the clinician.”(*directly quoting Jaeschke et al., 1989* [2] *even though ‘clinician judgment’ is not part of the original quote)*	• “Here, the terms ‘clinical importance’ and ‘clinical meaningfulness’ will be used interchangeably as an alternative to clinical significance because the word ‘significance’ has statistical connotations that obscure the issues at hand. Clinical importance is concerned with the question of whether a treatment results in real, meaningful, observed, or self-reported change in the patient’s life and functioning.” • With regard to the definition of MCID by Jaeschke et al. (1989), clarifies that “(…) the ‘difference’ in MCID refers to change within a single patient rather than a between-group comparison of means.” • Describes two types of minimal change, i.e., the ‘Detection Problem’ and the ‘Clinical Prediction Problem’, with the former seeking to answer “(…) whether the treatment effect observed by the chosen research variable is larger than that variable’s measurement error.”, and the latter seeking “(…) evidence for whether a research variable is a valid predictor of clinically meaningful changes in 1 or more desired patient outcomes.” • “(…) there is some abuse of terminology in the literature. This occurs most often when an MCID is reported as calculated through distributional methods. In this case, what is being calculated is in fact a measure of the Detection Problem, because the calculation relies only on the variance in the outcome and not its relationship to a clinical anchor.”
King et al., 2019 [46]	Terminology and methods of the MID critique, providing a historical context for the various ‘how to’-focused papers, which summarize methods and provide recommendations; article selection was not based on a systematic search	• Minimal clinically important difference (MCID) • Minimally important change (MIC) • Minimally important difference (MID) • Responder definition	• *MCID*: “The [MCID] was first defined as: ‘The smallest difference … which patients perceive as beneficial and which would mandate, in the absence of troublesome side effects and excessive cost, a change in the patient’s management’ (*quoting Jaeschke et al., 1989* [2]). • *MID*: “For clarity in this paper, we use *MID* to refer to the smallest clinically meaningful *difference between groups or individuals* (…)” • *MIC:* “(…) and *MIC* to refer to the smallest clinically meaningful *change over time within a group or an individual*.” • *Responder definition*: “a score change in a measure, experienced by an individual patient over a predetermined time period that has been demonstrated in the target population to have a significant treatment benefit.” *(quoting FDA PRO Guidance 2009* [21]).”	• “Several similar terms [*to minimal clinically important difference*] have emerged since, differing only slightly in definition and generally estimated and used in similar ways.” • With regard to terminology, authors make a distinction between “difference” and “change” over time; use the terms MID (for difference between groups or individuals) and MIC (for change over time within a group or an individual) and provide definitions for “clarity in this paper”.
Devji et al., 2021 [47]	To improve the understanding of MID concepts and awareness of common pitfalls in methodology and reporting to better inform the application of MIDs in clinical research and decision-making	• Minimal important difference (MID)	• *MID*: “the smallest change—either positive or negative—in an outcome that patients perceive as important” (*referencing Jaeschke et al., 1989* [2])	• “… inconsistencies in terminology add unnecessary complexity to reviewers’ task in comprehensively identifying relevant MIDs, requiring meticulous inspection of methodology in individual studies to ensure estimates offered truly reflect the MID. Thus, to avoid confusion and minimise the risk of MIDs going undetected when searching the literature, effort should be made to standardise terminology, and where appropriate reflect important conceptual distinctions” • “In 1987, Guyatt et al. introduced the MID concept, labelling the concept the minimal *clinically* important difference. Because this terminology focused attention on the clinical arena as opposed to patients’ experience, the same group of researchers later suggested dropping the word ‘clinical’, and relabelled the concept as the MID.” (emphasis in italics by original authors) • “In the 338 anchor-based MID studies currently included in our inventory, we identified 86 unique terms referring to the MID concept (…). Most deviations from the original and revised terminology were trivial (eg, *minimum clinically* important difference), but others were more problematic (eg, clinically relevant change, minimal patient perceivable deterioration, responder definition improvement). This profusion of terms is often a semantic matter, but sometimes represents a different concept. For instance, MIDs for improvement may sometimes differ from those of deterioration, and thus being explicit about the direction of the difference (or change) may be warranted (eg, minimal important improvement, minimal important deterioration). (…) Further, some researchers suggest the usefulness of distinguishing between methods that rely on within-person changes and those that quantify differences between groups.” (emphasis in italics by original authors) • “Some of the issues we have labelled—in particular, terminology and completeness of reporting, are easily addressed.”
Terwee et al., 2021 [48]	1) to clarify the concept of MIC and how to use it 2) to provide practical guidance for estimating methodologically sound MIC values 3) to improve the applicability of PROMIS by summarizing the available evidence on plausible PROMIS MIC values	• Mean change threshold • Minimal detectable change (MDC) • Minimal important change (MIC)* • Minimal important difference (MID) • Responder definition • Smallest detectable change (SDC) *Term used by authors	• *MIC:* “We define the MIC as a threshold for a minimal within-person change over time above which patients perceive themselves importantly changed. (…) The MIC does not refer to thresholds for changes that are considered more than minimal. (…) The MIC is not a minimal *detectable* change. (…) the MIC is not a *difference* between (groups of) patients.” (emphasis in italics by original authors) • *MID:* “(…) a difference between patients who reported to be ‘a little better’ and those who reported to be ‘about the same” refers to a minimal important *difference* (MID), not a minimal important within-person *change* (MIC).” (emphasis in italics by original authors) • *MDC*: “(…) (MDC, also referred to as [SDC]). The MDC is the smallest change in score (…) detected statistically with some degree of certainty (e.g., 95 or 90%), based on the standard error of measurement (SEM) or limits of agreement from a test–retest reliability design. The MDC does not relate to the importance of change to the patients under investigation.”	• “(…) a lot of confusion about the concept of MIC, (…) which questions the validity of published MIC values.” “First, there is inconsistency in terminology used (e.g., minimal important change, minimal important difference, minimal clinically important difference, meaningful change threshold, to name a few). Similar terms may refer to different concepts and vice versa.” • “Second, there is particular confusion about the concepts of minimal *important* change and minimal *detectable* change, which refer to different concepts.” (emphasis in italics by original authors) • “Third, there are differences in methods used for estimating the MIC, some more and some less methodologically sounds. This confusion hampers and may even bias the interpretation of PROM change scores in research and clinical practice.” • “Assuming that all patients have their individual threshold of what they consider a minimal important change, the MIC can be conceptualized as the mean of these individual thresholds. This definition of MIC is made up of three important elements: first, it refers to a threshold for a *minimal* change above which patients perceive themselves as changed (improved or deteriorated). Second, it refers to a change that is considered *important* to patients. And third, it refers to a within-patient *change* over time.” (emphasis in italics by original authors)
Trigg and Griffiths 2021 [49]	To review triangulation approaches and provide recommendations	• Meaningful change threshold (MCT)	• *MCT*: “(…) any score threshold that, when surpassed, indicates that a change or difference that is meaningful to patients has been observed.”	• MCT used as general term • “While terminology varies considerably (…), contemporary understanding distinguishes between-group differences from within-individual changes over time, although within-group changes are also recognised. Within the context of a randomised controlled trial, a between-group difference threshold should be used to interpret the differences in change over time between two treatment groups, whereas within-group change thresholds should be used to gauge the extent of improvement or worsening over time in a single treatment group. Within-individual change thresholds are commonly used to define responders and perform subsequent responder analysis.” • “(…) MCTs are not necessarily required to reflect a ‘minimally important’ amount of change, but can also guide the interpretation of larger changes in HRQoL where there is greater confidence that this is meaningful.” “Ultimately, the MCT supports the consequential validity of score inferences, where these may mandate a change in a patient’s care (based on within-individual change) or approval and reimbursement of novel therapies (based on between-group differences).” Note that, interestingly, Trigg and Griffiths (2021) provide a reference to Messick (1989) [50], making the link between Messick’s validity theory and consequences of testing and Jaeschke et al.’s (1989) ‘change in patient’s management’ (or *here:* ‘change in a patient’s care’).
Van der Willik et al., 2021 [51]	To provide insight into the interpretation of PROM scores by introducing the different types and characteristics of PROMs, and the most relevant concepts for the interpretation of PROM scores	• Minimal detectable change (MDC) • Minimal important change (MIC) • Minimal clinically important change • Minimal clinically important difference	• *MDC*: “The MDC is a parameter of reliability and is defined as the ‘smallest change in score that can be detected beyond measurement error’. Thus, the MDC reflects the threshold at which a change in score can be considered statistically significant.” • *MIC:* “(…) ‘the smallest change in score in the construct to be measured which patients perceive as important’.” *(referencing De Vet et al., 2011* [52])	• Notes the use of different terminology for the same concept: • “(…) the [MIC] or minimal clinically important change, in the literature also referred to as the minimal (clinically) important difference.” • “(…) the [MDC], also known as the smallest detectable change or the minimal real change.” • “(…) MIC gives an indication of what is *on average* considered important by an individual and should therefore be considered as a probability-threshold to interpret individual changes: if an individual change is larger than the MIC, the probability that this change is perceived important by the patient is greater than the probability that this change is perceived as not important.”
Zervos et al., 2021 [53]	To review the overall concept, method of calculation, strengths, and weaknesses of MCID and its application in the neurosurgical literature	• Minimal clinically important difference (MCID) • Minimally detectable difference (MDD)	• *MCID*: “(…) the smallest amount of symptomatic improvement or worsening that matters to a patient and influences treatment decisions.” *(referencing McGlothlin et al., 2014* [54]). • “Restated, it is the smallest degree of change that a person recognizes as different from their prior state, outweighing the costs and side effects of a treatment, warranting pursuit.” *(referencing Jaeschke et al., 1989* [2]*; Turner et al., 2010* [55]*; Wells et al., 2001* [56]) • *MDD*: “(…) a change beyond the 95% CI has been termed minimal detectable difference (MDD) or minimal detectable change and serves as a proxy for MCID.” *(referencing Turner et al., 2010* [55]*; Beckerman et al., 2001* [57])	• MCID generally used broadly by authors, with no distinctions in concepts, such as individual or group level, or change versus difference. For example: “Over 9 methods have been proposed to determine MCID, the most common being consensus, anchor, and distribution techniques.” • “MCID is a floor value and not an optimal change. When optimal change is of interest, then a separate clinimetric tool such as the ‘substantial clinical benefit’ may be preferable.”
Kolin et al., 2022 [58]	To review MCID quantification and reporting in existing studies on shoulder arthroplasty	• Minimum clinically important difference (MCID) • Substantial clinical benefit (SCB) • Patient acceptable symptom state (PASS)	• *MCID:* “(…) the smallest difference in an outcome measure that a patient would perceive as a meaningful change.” *(among others, referencing Jaeschke et al., 1989* [2]) • *SCB:* “(…) the value where patients exceed the minimum threshold of improvement or report achieving a substantial clinical benefit.” *(referencing Glassman et al., 2008* [17]) • *PASS:* “(…) a PROM target value beyond which patients consider themselves to have reached an acceptable outcome.” *(referencing Kvien et al., 2007* [59])	• Used MCID as a general term, making no distinctions between group-level or individual-level changes or differences, or method used for estimation • “Previous authors have argued that the SCB should be (…)target level of improvement, whereas the MCID is a floor threshold.” • “PASS is considered to be representative of patient satisfaction (…).”
Leidy et al., 2022 [60]	1) to clarify the history, terminology, and methods related to development of score interpretation thresholds 2) to apply this information to the E-RS:COPD	• Minimal clinically important difference (MCID) • Minimal important difference (MID) Responder definition	• *MCID:* “(…) smallest difference (change) in the domain score of interest which patients perceive as beneficial and which would mandate, in the absence of troublesome side effects and excessive cost, a change in the patient’s management.” *(quoting Jaeschke et al., 1989* [2]) • *MID*: “In the 1990s, questions were raised about the extent to which a patient rating could be labeled a *clinical* variable; reference to clinical was dropped, resulting in the phrase ‘minimal important difference’ (MID) to refer to meaningful within-patient change, and later to within-group and between-group differences.” *(referencing Juniper et al., 1994* [43]*; Schünemann et al., 2005* [61]*; Wyrwich et al., 2013* [62]; emphasis in italics by original authors) • *Responder Definition:* “Meaningful change in an *individual’s response to treatment* (…)” *(referencing FDA Draft PRO Guidance* [44]; emphasis in italics by original authors)	• “MID, or the occasional reference to the MCID, refers to group-level data (within-group change and between-group differences) that can be interpreted as meaningful based on empirical evidence consistent with the context of use.” MID values are expressed as numeric summaries (e.g., mean) and are often used to inform power estimates for statistical analyses of between-group differences during trial design. • “In contrast, a responder definition refers to patient-level data, and is the threshold of within-patient change in the PRO score that can be interpreted as meaningful, again based on empirical evidence consistent with the context of use.” • “For hypothesis testing to uncover and understand treatment effects, a 2-step process is often used, with group-level comparisons powered using the pre-specified MID estimate and tested statistically, followed by descriptive responder analyses to assist with interpretation when group-level effects are statistically significant”
Speeckaert et al., 2022 [63]	To evaluate how published MID values for ClinROs should be interpreted (with focus on dermatology)	• Minimal important difference (MID) • Minimal clinically important difference (MCID)	• *M(C)ID*: “(…) defined as the smallest difference in an outcome measure that is perceived to be important by patients.” *(referencing Salas Apaza et al., 2021* [64]) • *MID:* “(…) ‘the smallest difference that would mandate, in the absence of troublesome side effects and excessive cost, a change in the patient’s management’.” *(quoting Jaeschke et al., 1989* [2])	• To our knowledge, one of the first publications that systematically explores MIDs for ClinROs (*here:* restricted to skin disorders) • Use MID as general term, making no distinctions between group-level or individual-level changes or differences • “Although there has been debate about what constitutes an ‘important’ difference, the most widely accepted definition of the MID remains the statement of Jaeschke et al. 1989, (…).” • “Some authors prefer different terms than the MID, such as ‘clinically important change’ or ‘meaningful change’. Given the differences in terminology and in methods to determine the MID, the interpretation of MID values can become confusing and even misleading. This may result in wrong conclusions about the efficacy of treatments.” • “In 12/29 (41.4%) cases, the MIDs for ClinROMs represented a substantial difference in disease severity assessed by physicians. This violates two basic assumptions from the definition of the MID by Jaeschke et al., namely, that the difference should be minimal and is based on the experience of the patient. (…) From the eight studies that did not include the word ‘minimal’ but terms such as ‘meaningful change’ or ‘clinically important difference’, four studies (50%) did assess a minimal difference.”
Wang et al., 2022 [65]	To systematically explore methodological issues in studies estimating anchor-based MIDs	•Minimal important difference (MID)	• *MID*: “(…) a threshold that represents the smallest important difference or change, either beneficial or harmful, that patients perceive as important (…).”*(referencing Jaeschke et al., 1989* [2]*; Schünemann et al., 2005* [61])	• Used MID as a general term, making no distinctions between group-level or individual-level changes or differences • “(…) most articles that offered a definition (65 out of 95) [defined] MID as ‘the smallest difference in score in the outcome of interest that informed patients or informed proxies perceive as important, either beneficial or harmful’, representing a modification of the original definition by deleting the phrase ‘in the absence of troublesome side-effects and excessive cost, a change in patient management’.”
Mabrouk et al., 2023 [66]	To review MCID and PASS in terms of definition, calculation, clinical application and relevance, and limitations	• Minimal clinically important difference (MCID) • Minimal clinically important improvement (MCII) • Patient acceptable symptom state (PASS)	• *MCID:* “(…) ‘the smallest difference in score in the domain of interest, which patients perceive as beneficial and which would mandate, in the absence of troublesome side effects and excessive cost, a change in the patient’s management’.” *(quoting Jaeschke et al., 1989* [2]) • *MCII: “A similar measure, which (…) focuses only on a positive change*.” *(referencing Kvien et al., 2007* [59]) • *PASS: “(…)* a level of symptoms or degree of functioning that the patient would describe as acceptable should they remain at that level.” *(referencing Kvien et al., 2007* [59])	• Use MCID as general term, making no distinctions between group-level or individual-level changes or differences, or method used for estimation • “To simplify both measures, MCID is used to evaluate whether a patient feels ‘better/worse’ after the treatment, while PASS assesses whether a patient feels ‘well’ at a given time point.” • “[For PASS] the patient has achieved a state of symptoms that they can be satisfied with in the longer term.”
Mishra et al. 2023 [67]	To review MCID definitions, methods, thresholds and their limitations in neurological conditions	• Minimal clinically important difference (MCID)	• *MCID:* “(…) ‘the smallest change in the outcome measure that patients perceive as beneficial’” (*quoting Sloan, 2005* [68]). AND “(…) ‘smallest difference in score in the domain of interest which patients perceive as beneficial and which would mandate, in the absence of troublesome side effects and excessive cost, a change in the patients’ management’.”(*quoting Jaeschke et al., 1989* [2])	• Use MCID as general term, making no distinctions between group-level or individual-level changes or differences, or method used for estimation • “MCID and its inconsistent nomenclature include MID, minimal MCIC, CID, and meaningful change.” • “MCID values are important in interpreting the clinical relevance of observed changes, at both individual and group levels, and can be patient or clinician centred.”
Tanghe et al., 2023 [69]	To review quantitative metrics for assessing clinical improvement with PROMs following total hip arthroplasty	• Minimal clinically important difference (MCID) • Minimal clinically important improvement (MCII) • Minimal detectable change (MDC) • Patient acceptable symptom state (PASS) • Substantial clinical benefit (SCB)	• *MCID*: “(…) ‘the difference in score in the domain of interest which patients perceive as beneficial and which would mandate, in the absence of troublesome side effects and excessive cost, a change in the patient’s management.” (*quoting Jaeschke et al., 1989* [2]) • *MCII: “(…)* measure that represents the concept of improvement or ‘feeling better’.” *(referencing Tubach et al., 2012* [70]) • *MDC:* “(…) the minimal change that can be detected utilizing the standard error of measurement (SEM).” *(referencing Copay et al., 2018* [71]) • *PASS: “(…)* a threshold value of a given PROM beyond which a patient considers themselves well, or ‘feeling good’ (…)”*(referencing Tubach et al., 2012* [70]) • *SCB*: “(…) threshold value of a given PROM for substantial improvement (‘much better’)” *(referencing Cvetanovich et al., 2019* [72]*; Su et al., 2021* [73])	• “It [MCID] represents the threshold for a change meaningful and worthwhile to the patient to feel ‘somewhat better’ if an intervention was to take place. Any value above this calculated value would be considered clinically important and could indicate a valuable intervention.” • “MCII is a closely related measure that represents the concept of improvement or ‘feeling better’.” • “(…) MDC is purely statistical. (…) It is the minimal change in a PROM score that falls outside the measurement error in the score of an instrument used to measure a given symptom. Thus, it can be useful to assess the reliability of a calculated MCID value.” • “This distinction *[with PASS]* is important, as patients can report a perceived change in state without necessarily feeling that they have reached an acceptable state of improvement, a flaw in many of the other metrics.” • “SCB represents (…) substantial improvement (‘much better’), indicating success following intervention rather than minimal improvement like MCID (‘somewhat better’).”
Shaw et al., 2024 [74]	To identify to what extent information on the MID of the EQ-5D is required and used by selected HTA agencies	• Minimally important difference (MID) • Minimal important change (MIC)	• *MID:* “(…) ‘the smallest difference in score in the domain of interest that patients perceive as important, either beneficial or harmful, and which would lead the clinician to consider a change in the patient’s management.” *(quoting Guyatt et al., 2002* [33])	• Use MID as a general term • “Terminology relating to MID can be confusing, with multiple terms that differ in definition, which have led to inconsistency in terminology used.” “De Vet and Terwee (2010) highlight that while MIC and MID are frequently used interchangeably, the authors prefer the use of MIC instead of MID, in order to differentiate changes from differences.” • *[Based on review of Technology appraisal (TA) documents published between 1 January 2019 and 15 January 2021 from HAS, IQWiG, G-BA, NICE, and ICER]* “While terminology for MID was variable (minimal(ly) important difference, minimal clinically important difference, clinically meaningful, clinically meaningful difference, clinically meaningful change, clinically meaningful improvement, clinically relevant improvement, clinically relevant deterioration), the term ‘minimal(ly) important difference’ was most frequently reported, in 72% of TAs mentioning MID (*n* = 42/58). However, limited explanation of the methodology utilised made it difficult to assess whether the correct terms were employed. In contrast to Terwee et al.’s 2021 definition of MIC as longitudinal and MID as cross-sectional, the G-BA considered MID as longitudinal.”

A consistent theme reported across the 16 review articles was the confusion around terminology in the literature, the lack of consistency of terms used, and the negative consequences of using the same terms to refer to different concepts or of not clearly defining terms. The reviews, having been published two decades or more after the introduction of the term “MCID”, continued to highlight a need for clear and consistent terminology and a call to action to address this need [47, 48]. While Devji et al. (2021) [47] remarked that the “terminology problem” is one that is “easily addressed”, it is clear that harmonizing terminology has been, and continues to be, a challenge.

The methodology review articles highlight that inconsistent terminology has consequences for interpretation of COA scores and can lead to uncertainty about how score interpretation thresholds should be applied and whether conclusions drawn about treatment benefit based on these thresholds are appropriate [47, 48]. Several papers noted interchangeability of different terms in the literature, such as MCID, MID, and MIC [63, 67, 74]. In some cases, the authors did not make judgments about whether the terms were appropriate or if distinctions should have been made between terms [51, 53]. Other articles sought to clarify definitions and explain the distinctions between terms [20, 45, 47, 48]. In some review articles, authors described erroneous uses of terms in the literature, such as MID incorrectly being used for both individual-level and group-level change [20] or MCID incorrectly being used for thresholds derived based on distribution-based methods rather than only anchor-based methods [45].

Despite most articles pointing out similar issues related to the inconsistent use of terms and definitions, the clarifications and distinctions between terms provided were not always consistent across review papers either. For example, distinctions between MCID and MID were sometimes made based on whether the term reflected a difference at the group level or individual level [45, 48], and sometimes whether ‘importance’ required clinical judgment [20, 47]. Even in the latter case, there were differences in interpretation, with some suggesting that the removal of the word ‘clinical’ from MCID indicated that MID is focused on the patient’s perspective and does not require the clinician’s judgment of importance [20, 47], while other definitions of MID still included the term “clinically” to qualify “meaningful” when defining MID [46]. One paper indicated that the MID is derived statistically using distribution-based methods and, therefore, does not take into account what patients or clinicians consider to be important [45].

Several review articles used a preferred term as a general term or with a clear definition to avoid confusion. For example, the term ‘minimal important change (MIC)’ was used by Terwee et al. (2021) [48], and a comprehensive definition was provided explaining three components: 1) the threshold represents a minimal change, above which would be considered an important amount of change (improved or deteriorated); 2) importance is from the perspective of the patient; and 3) the threshold refers to within-person change over time. King et al. (2019) [46] used the term MID to refer to differences between groups or individuals and MIC to refer to changes over time within a group or individual, and defined them specifically in this way to avoid confusion. Others provided recommendations for terms in an effort to harmonize terminology, including introducing new terms or suggesting that certain historically confusing terms, such as MCID and MID, be avoided [20].

Overall, confusion related to terminology and recommendations for clarification generally fell into four main areas:

- Perspective: Which/whose perspective is considered important (e.g., patient, clinician, statistical)?
- Magnitude: What level of difference/change does the threshold represent (e.g., minimal, detectable, important, meaningful, substantial)?
- Application: How should the threshold be applied (i.e., intended use of the interpretation threshold)?
  Individual level versus group level
  Cross-sectional ‘difference’ versus longitudinal ‘change’
  Improvement versus deterioration
- Methodology: What methodology was used to derive the threshold (e.g., anchor-based, distribution-based, qualitative)?

#### Perspective

Terms were generally defined either as representing changes/differences that were important to patients (with or without the judgment of clinicians), or were important statistically, meaning that the difference/change was larger than measurement error but not necessarily ‘important’ to patients. Comments on the meaning of the term ‘clinical’ and whether it should be included in the term were often provided, though explanations of the meaning and use of ‘clinical’ were not always consistent.

#### Magnitude

Comments on terms used to define the level of change/difference of the threshold were common, such as the meaning and use of ‘minimal’ and the distinction between changes that are ‘detectable’ versus ‘important’. Zervos et al. (2021) [53] commented that the MCID represents a ‘floor’ value rather than optimal change, while Coon and Cappelleri (2016) [20] noted that noticeable changes are not necessarily meaningful. Distinctions in magnitude for different terms were sometimes clarified using anchor levels, such as “much better” to clarify the magnitude of “substantial improvement” versus “somewhat better” indicating “minimal improvement like MCID” [69].

#### Application

When defining terms, clarification on how the threshold should be applied was often provided, and the proposed application was sometimes linked to the method used to derive the threshold. For example, sometimes thresholds derived using distribution-based methods were reported as applicable only for group-level comparisons. Distinctions were commonly noted as to whether a term applied specifically to group-level differences/changes, individual-level differences/changes, or both, but distinctions were inconsistent. For example, Coon and Cappelleri (2016) [20] noted that MID was used to interpret group-level mean differences, whereas King et al. (2019) [46] defined MID as being applicable to “differences between groups or individuals”. Sometimes distinctions were made as to whether a threshold term applied to cross-sectional differences (e.g., MID) versus longitudinal changes over time (e.g., MIC), regardless of whether differences/changes were examined at the individual or group level [46].

Conversely, others placed emphasis on whether the threshold was applied to groups or individuals, regardless of whether examining cross-sectional differences or longitudinal changes over time [20]. Finally, it was noted that interpretation thresholds may differ for improvement and worsening. For this reason, Devji et al. (2021) [47] recommended clearly indicating the direction for which the threshold applies (e.g., minimal important improvement versus minimal important deterioration).

#### Methodology

Several papers linked specific terms to specific methods for estimating score interpretation thresholds, suggesting that terms are sometimes used erroneously based on the method used. Collins (2019) [45] reported that MID and MDC describe thresholds that are larger than measurement error (i.e., would be estimated using distribution-based methods), whereas MCID and MIC require anchor-based methods for estimation. Shaw et al. (2024) [74], commenting on the different terms used in HTA reviews, noted difficulty in determining whether the correct term was used due to insufficient methodological detail.

### Terms across different stakeholders, focusing on regulatory authorities and HTA/Payers

In Table 2, eight regulatory and HTA/payer agencies were identified with published guidance documents that included terminology describing COA score interpretation thresholds, including two regulatory agencies (FDA [21, 44, 75], EMA [76, 77]) and six HTA/payer organizations: Institute for Quality and Efficiency in Health Care (IQWiG, Germany) [78], Canadian Agency for Drugs and Technologies in Health (CADTH, Canada) [79], Medical Services Advisory Committee (MSAC, Australia) [80], Pharmaceutical Benefits Advisory Committee (PBAC, Australia) [81], Haute Autorité de Santé (HAS, France) [82], and HTA CG [83]. The use of terminology was generally inconsistent, with different terms used within and across agencies. MID and MCID were most used to describe score interpretation thresholds. Other terms used included responder definition, response threshold, response criterion, within-patient change threshold, individual minimally important difference, PASS, clinical relevance threshold, meaningful score difference (MSD), and meaningful score region (MSR).

**Table 2:** Overview of terms describing score interpretation thresholds used by government agencies, regulatory bodies, and payers

Agency/Reference	Source(s)	Terms Used for Score Interpretation Threshold	Excerpts with Terms and Definitions
REGULATORY			
FDA [44]	Guidance for industry: patient-reported outcome measures: use in medical product development to support labeling claims: draft Guidance (2006, DRAFT)	• Minimum Important Difference (MID)	• *MID*: “Amount of difference or change observed in a PRO measure between treatment groups in a clinical trial that will be interpreted as a treatment benefit.”
FDA [21]	Guidance for Industry Patient-Reported Outcome Measures: Use in Medical Product Development to Support Labeling Claims (2009)	• Responder Definition	• *Responder Definition*: “A score change in a measure, experienced by an individual patient over a predetermined time period that has been demonstrated in the target population to have a significant treatment benefit” • “Regardless of whether the primary endpoint for the clinical trial is based on individual responses to treatment or the group response, it is usually useful to display individual responses, often using an a priori responder definition (i.e., the individual patient PRO score change over a predetermined time period that should be interpreted as a treatment benefit)” • Other comments related to terminology and definitions: • “*clinically meaningful* (i.e., … indicate treatment benefit)” • “The empiric evidence for any responder definition is derived using anchor-based methods … Distribution-based methods for determining *clinical significance* of particular score changes should be considered as supportive and are not appropriate as the sole basis for determining a responder definition.”
FDA [75]	Patient-Focused Drug Development: Incorporating Clinical Outcome Assessments Into Endpoints for Regulatory Decision-Making (2023, DRAFT) - Guidance 4 -	• Meaningful Score Difference (MSD) • Meaningful Score Regions (MSR)	• “… two approaches to support interpretability of COA scores–interpreting in terms of **meaningful score differences** and in terms of **meaningful score regions**” • *MSD*: “What size difference between any two COA scores would be viewed as meaningful for patients.” • “Often, **MSD** is determined based on what patients would regard as a clinically meaningful within-patient change (i.e., improvement or deterioration from the patient’s perspective).” • “A range of MSD should be selected that reflects most patients.” • “MSD can be used in at least two ways: (1) to evaluate the expected treatment effect for the average patient in some target population; or (2) to use as a threshold in descriptive analyses that identify individual patients who might have changed by a meaningful amount.” • “Distribution based methods (e.g., effect sizes, certain proportions of the standard deviation and/or standard error of measurement) do not directly consider the patient voice, and as such, are insufficient to serve as the sole basis for identifying an MSD.” • *MSR*: “Another approach for interpreting the meaningfulness of treatment effect is to specify the meaning of individual COA scores so that it is easier to judge whether two or more scores (e.g., treatment group means at a prespecified time point) correspond to distinct health-related experiences of patients (…) meaningful score regions denote groups of scores that are thought to be similar to one another and different from other groups of scores in terms of the patient’s experience of the symptom(s) measured by the COA” • Other comments related to terminology and definitions: • “Knowing how COA scores relate to patients’ experiences is central to interpreting the meaningfulness of a COA-based endpoint result(s)”. “Note that ‘differences in COA scores’ is used here as a general term that includes differences that occur over time within a patient, i.e., changes in COA scores” • “Note that the roles of MSD or MSRs differ depending upon the type of endpoint. For endpoints based on continuous COA scores, the MSD or MSRs *help to interpret the treatment effect*. For this application, the sponsor can prespecify a range of MSD or MSRs that will be used to aid interpretation. For endpoints based on categorizing COA scores (e.g., a “responder” endpoint), the MSD or MSRs define the endpoint. In that case, the sponsor should prespecify a single threshold (for MSD) or set of thresholds (for MSRs) that will be used *to define the endpoints*.” • “The status could be defined based on an MSD approach by classifying patients’ changes from baseline (e.g., as “observed improvement,” “observed worsening,” “no change”). The endpoint could also be defined using a MSRs approach (e.g., patients scoring below some thresholds are classified as “symptoms resolved” and those scoring at or above the threshold are classified as “symptomatic”). For these situations, the sponsor should prespecify the threshold (in the case of MSD) or set of thresholds (in the case of MSRs) that will be used to define the endpoint.”
EMA (2005) [76]	Reflection paper on the regulatory guidance for the use of health related quality of life (HRQL) measures in the evaluation of medicinal products	• Minimal Important Difference (MID)	• “The **minimal important difference (MID)** may be used when powering the studies. It should be kept in mind, however, determination of **MID** should be based upon a combination of statistical reasoning and clinical judgment and neither alone is sufficient.”
EMA (2014) [77]	Reflection paper on the use of patient reported outcome (PRO) measures in oncology	• Minimal Clinical Important Difference (MCID)	• “The **Minimal Clinical Important Difference (MCID)** has been described as ‘the smallest difference in score in the domain of interest which patients perceive as beneficial and which would mandate, in the absence of troublesome side effects and excessive cost, a change in the patient’s management’.”
HTA/PAYER			
IQWiG, Germany (2023) [78]	General Methods; Version 7.0; 19 September 2023	• Response Threshold/Criterion • [Individual] Minimally Important Difference (MID)	• “… a **response threshold** reflects changes that are perceptible to patients with sufficient certainty.” • “In recent years, responder analyses based on a response criterion in terms of an **individual minimally important difference (MID)** have been increasingly conducted. However, the methodological problems of this approach are becoming increasingly apparent.” • “If responder analyses using an **MID** are pre-specified in a study and the **response criterion** corresponds to at least 15% of the scale range of the measurement instrument used, these responder analyses are used for the assessment without further examination of the **response criterion** … If pre-specified **response criteria** in terms of an **MID** are below 15% of the scale range, these are as a rule not used.”
CADTH, Canada (2025) [79]	Reimbursement Review: Pharmaceuticals With Anticipated Comparable Efficacy and Safety (PACES) Tailored Review Sponsor Submission Template (*was:* Tailored Review Sponsor Submission Template)	• Minimal Important Difference (MID) • Responder definitions	• “Descriptions of scale measures should include a brief overview of the scale including: (…) Whether or not an estimated **MID** was identified (for overall and individual domain scores). Please clearly state the source of the MID (e.g., reference to publication, regulatory opinion, clinical expert opinion) and the method used for estimation (e.g., anchor-based) and whether the MID refers to **within-group or between group differences** (or both). Identify the population in which the MID was estimated (e.g., patients with severe COPD; general population estimate). If multiple estimates of the MID are identified, the full range of MIDs should be reported. If no MID has been identified, this should be explicitly stated.” • “**Responder definitions**, cut points and rationale for cut point selection should be described and referenced.” Note that CADTH released a new template when this article was being processed for publication. Interestingly, the old template used the term ‘minimal clinically important difference’, whereas the new template now refers to MIDs and responder definitions.
MSAC, Australia (2021) [80]	Guidelines for Preparing Assessments for the Medical Services Advisory Committee; Version 1.0, May 2021	• Minimal Clinically Important Difference (MCID)	• “The definition of a **minimal clinically important difference (MCID)** varies across the literature. The central concept of an **MCID** is that it represents the smallest amount of difference in a score that would, in some way, be considered important. MCIDs may be reported in either relative or absolute measures.” • “Typically, an **MCID** reflects the average minimal difference in a score that is considered to be clinically important.” • “Study results are most commonly aggregated to reflect the average estimate of change in a score for each study arm. If the average change experienced by a study arm is lower than the **MCID** does not mean that, for some patients, a clinically important change has not occurred. Equally, if one arm reports an average change above the **MCID** and the other arm reports an average change below the **MCID**, it may not be possible to infer that the difference between the arms is clinically meaningful.” • “A more meaningful method of examining response is to report the proportion of participants who experienced a change in a score that was greater than the **MCID**. A responder analysis can also be represented by a cumulative distribution function such that the proportion of responders can be viewed across multiple thresholds for an **MCID**.”
PBAC, Australia (2016) [81]	Guidelines for preparing a submission to the Pharmaceutical Benefits Advisory Committee Version 5.0 September 2016	• Minimal clinically important difference (MCID) • Responder threshold	• “An **MCID** is the smallest difference in a particular outcome that patients perceive as beneficial (or detrimental). This is usually determined by patients, although an **MCID** may be determined by a consensus of experts.” • “For patient-relevant outcomes that are measured on a scale (e.g., a patient-reported outcome measure, a quality-of-life instrument, the Visual Analogue Scale, the LogMAR vision acuity test, the 6-Minute Walk Distance test) … the **MCID** can be used as a **threshold**, beyond which an individual patient would be regarded as a **‘responder’”**
HTA CG [83]	Originally EUnetHTA 21 – Individual Practical Guideline Document D4.4 – OUTCOMES (ENDPOINTS) Version 1.0, 25/01/2023 Template version 1.0, 03/03/2022; updated: Guidance on outcomes for joint clinical assessments; adopted on 10 June 2024 by the HTA CG pursuant to Article 3(7), point (d), of Regulation (EU) 2021/2282 on Health Technology Assessment	• Minimal Important Difference (MID) • Minimal Clinically Important Difference (MCID) • Within-patient change threshold • Responder definition Patient Acceptable Symptomatic State (PASS)	• “In general, a threshold, called a **responder definition,** can be used to classify whether or not a patient has experienced an improvement or deterioration of his or her condition. This can be done either by assessing whether or not a patient reached a pre-specified level of success, or by assessing whether the change in scores is at least equal to a pre-specified threshold.” • “The patient’s perspective is frequently used by linking a change in score to the subjective meaning of what is a relevant change according to patients. This approach is called the **minimal important difference (MID)** and can be defined as the minimal change in score perceived as an improvement or deterioration by the patient. This is also frequently called the **minimal clinically important difference (MCID),or meaningful change**. Although the term **MID** has been used to describe a threshold to interpret between-group differences in scores (e.g., difference in mean change from baseline), we use it within this guideline to refer to a **threshold** for interpreting **within-patient change** over time” “Another possible **responder definition**, albeit less common, is the concept of **patient acceptable symptomatic state (PASS)**, mostly used in rheumatology. Instead of focusing on the change in score that is perceived as beneficial by patients, the idea is to find the minimum score above which patients consider their health state as acceptable.”
HAS, France (2020) [82]	HAS Transparency Committee doctrine Principles of medicinal product assessments and appraisal for reimbursement purposes; 2 December 2020	• Clinical relevance threshold	• “In addition to efficacy and safety data and depending on the medical context, if an improvement in quality of life is demonstrated, this could lead to a CAV *[clinical added value]* level of higher than V in situations in which this finding is based on: ‒ the use of validated scales appropriate to the objective (preferentially specific); ‒ a rigorous methodology: objective and **clinical relevance threshold** pre-specified in the protocol, double-blind conditions, management of multiplicity of the analyses, appropriate analysis frequency, time and duration, few missing data.”

*Other agency guidance documents reviewed, but no mention of score interpretation threshold identified:

• Japan: Pharmaceuticals and Medical Devices Agency (PMDA) 07 September 2021 [84]

• Japan: Center for Outcomes Research and Economic Evaluation for Health, National Institute of Public Health (C2H) Guideline for Preparing Cost-Effectiveness Evaluation to the Central Social Insurance Medical Council—Version 3.0 approved by CSIMC on 19 January 2022 [85]

• UK: National Institute for Health and Care Excellence (NICE) health technology evaluations: the manual, Process and methods; Published: 31 January 2022 [86]

• US: Institute for Clinical and Economic Review (ICER) 2020–2023 Value Assessment Framework; 31 January 2020 (Updated 23 October 2020) A Guide to ICERs Methods for Health Technology Assessment; 27 October 2020 [87]

• Scotland: Scottish Medicines Consortium (SMC): Working with SMC – A Guide for Manufacturers; March 2023 [88]

• Sweden: Pharmaceutical Benefits Board: General guidelines for economic evaluations from the Pharmaceutical Benefits Board (LFNAR 2003:2); April 2003 [89]

FDA and EMA published multiple guidance documents that included score interpretation threshold terminology. The terms used to describe these thresholds changed over time and differed between the two agencies. In the case of FDA, terminology evolved from the initial Draft PRO Guidance for Industry published in 2006 [44] to the most recent patient-focused drug development (PFDD) guidance series [75, 90–92]. In the 2006 Draft PRO Guidance for Industry, FDA introduced the term ‘minimal important difference (MID)’ and defined it as “(…) the amount of difference or change observed in a PRO measure between treatment groups in a clinical trial that will be interpreted as a treatment benefit” [44]. In the final version of this guidance, published three years later, FDA omitted the term MID and introduced the concept of ‘responder definition’, thus shifting emphasis from thresholds to interpret group mean score differences to thresholds to interpret change over time at the individual patient level [21].

In 2023, FDA issued the PFDD Draft Guidance 4 [75], which expanded beyond the concept of the responder definition and introduced two novel terms, i.e., meaningful score difference and meaningful score region. Substantial commentary on the meaning of these terms was provided, along with guidance on how they should be applied. MSD was defined as the “(…) difference between any two COA scores that would be viewed as meaningful for patients”. In contrast, MSR specifies the meaning of individual scores in terms of the patient’s experience (e.g., score ranges that represent mild, moderate or severe symptoms). It was noted that the MSD and MSR can be applied at the individual or group level and in different ways depending on the type of endpoint. For both MSD and MSR, anchor-based methods would be required to derive the thresholds because ‘meaningful’ is based on what patients consider meaningful. Of note, Guidance 4 is still in draft form and has received extensive commentary by various stakeholder groups. A common theme in the comments submitted to FDA was a concern that introducing the terms MSD and MSR would cause even more confusion in the field. It remains to be seen if the final Guidance 4 will include changes to the terms suggested in the draft Guidance document.

Like FDA, EMA first introduced the term MID in its 2005 Reflection Paper on the use of HRQoL measures in the evaluation of medical products [76]. EMA noted the use of MID for powering studies and advised that MID should be determined based on both statistical importance and clinical judgment [76]. In 2014, EMAs draft reflection paper on the use of PROs in oncology switched to the term MCID [77] and defined it using the original definition put forth by Jaeschke et al. (1989) [2].

Across guidelines published by the six HTA/Payer organizations identified in our search, all but one used the terms MCID and/or MID. MSAC acknowledged that definitions of MCID have varied in the literature [80], and HTA CG indicated that MCID and MID are used interchangeably. IQWiG, MSAC, PBAC, and HTA CG suggested that the MCID/MID can be applied at the individual level as a ‘responder’ threshold [78, 80, 81, 83]. HTA CG also referenced the use of the ‘Patient Acceptable Symptomatic State’ – already briefly introduced in our Background section – defined as the “(…) minimum score above which patients consider their health state as acceptable”, as an alternative responder definition and indicated it has mostly been used in rheumatology [83]. HAS did not specifically define a score interpretation threshold but referred to the ‘clinical relevance threshold’, which must be pre-specified for consideration as clinical added value [82]. PBAC indicated that MCID, while generally determined by patients, may be based on expert consensus [81].

### Review of use of score interpretation thresholds as part of articles aimed at deriving score interpretation thresholds

After removing duplicates and articles that did not describe the derivation of thresholds, 318 articles were identified for inclusion. Specific terms used to describe thresholds in these articles were summarized overall and by therapeutic area (TA). The articles were categorized into 11 TAs, including an ‘other’ category. Forty-one percent of the terms identified were in musculoskeletal condition papers (*n* = 174), followed by autoimmune/allergy (*n* = 58), other (*n* = 45), and neurology (*n* = 33). Fewer than 10 terms were identified in the categories of cardiovascular and mental health condition papers.

Among the 318 papers reviewed, 39 different terms were used to describe COA score interpretation thresholds (Table 3). MCID was the most used term overall, appearing in just over half of the articles (163) and in all 11 TAs. MID was the second most used term, appearing in 24% of papers, and in the TAs of oncology, women’s health, and mental health, MID was the most used term. Notably, ‘minimal important change (MIC)’ was the second most used term (after MCID) in the musculoskeletal category as opposed to MID. Other terms used in musculoskeletal conditions that were not or rarely included in other TAs were PASS and SCB. Thirty-two of the 39 terms (82%) were used in fewer than 10 papers, with 21 of these used only once.

**Table 3:** Overview of terms used across 318 papers aimed at deriving thresholds published between 2016 and 2021

		Therapeutic area											
		Autoimmune/ allergy	Cardiovascular	Mental health	Musculo- skeletal	Neurology	Oncology	Pain	Rare	Respiratory	Women’s health	Other	Total
Abbreviation	Term	(*n* = 58)	(*n* = 9)	(*n* = 6)	(*n* = 174)	(*n* = 32)	(*n* = 29)	(*n* = 11)	(*n* = 20)	(*n* = 28)	(*n* = 16)	(*n* = 45)	(*n* = 428)
MCID	Minimal Clinically Important Difference	14	4	1	71	16	12	4	8	16	4	13	163
MID	Minimal Important Difference	11	2	4	12	5	13	1	3	9	6	10	76
MIC	Minimal Important Change	8	0	0	32	3	0	0	0	1	2	8	54
SDC	Smallest Detectable Change	7	0	0	11	0	0	0	0	0	1	2	21
MDC	Minimal Detectable Change	0	1	0	9	2	0	1	1	1	0	4	19
PASS	Patient Acceptable Symptom State	2	0	0	13	1	0	0	0	0	1	0	17
SCB	Substantial Clinical Benefit	0	1	0	15	0	0	0	0	0	0	0	16
CID	Clinically Important Difference	2	1	0	3	0	0	0	2	0	1	0	9
CMC	Clinically Meaningful Change	2	0	0	0	0	1	0	1	1	0	3	8
MCIC	Minimal Clinically Important Change	0	0	0	3	0	0	1	0	0	1	0	5
CIR	Clinically Important Response	0	0	0	1	0	0	0	2	0	0	0	3
MCII	Minimum Clinically Important Improvement	1	0	0	0	0	0	1	0	0	0	1	3
RD	Responder Definition	0	0	0	0	1	2	0	0	0	0	0	3
CMI	Clinically Meaningful Improvement	1	0	0	0	0	0	0	0	0	0	1	2
MCT	Meaningful Change Threshold	1	0	0	0	0	0	0	1	0	0	0	2
MWC	Meaningful Within-Person Change	1	0	0	0	0	0	0	1	0	0	0	2
MWPC	Meaningful Within-Patient Change	1	0	0	0	1	0	0	0	0	0	0	2
-	Threshold For Meaningful Change	2	0	0	0	0	0	0	0	0	0	0	2
ICSD	Ideal Clinically Significant Difference	0	0	0	0	0	0	1	0	0	0	0	1
MCIW	Minimum Clinically Important Worsening	1	0	0	0	0	0	0	0	0	0	0	1
MCRC	Minimal Clinically Relevant Change	0	0	0	0	1	0	0	0	0	0	0	1
MCSD	Minimum Clinically Significant Difference	0	0	0	0	0	0	1	0	0	0	0	1
MDD	Minimal Detectable Difference	0	0	0	0	0	0	0	0	0	0	1	1
PPAA	Patient Perceived Adequate Analgesia	0	0	0	0	0	0	1	0	0	0	0	1
RID	Really Important Difference	0	0	0	1	0	0	0	0	0	0	0	1
SDD	Smallest Detectable Difference	1	0	0	0	0	0	0	0	0	0	0	1
SMD	Standardized Mean Difference	0	0	0	1	0	0	0	0	0	0	0	1
SRC	Smallest Real Change	0	0	0	0	1	0	0	0	0	0	0	1
-	Clinically Important Change	0	0	0	0	0	0	0	0	0	0	1	1
-	Clinically Important Threshold	0	0	0	1	0	0	0	0	0	0	0	1
-	Clinically Meaningful Responder Threshold	1	0	0	0	0	0	0	0	0	0	0	1
-	Important or Meaningful Change	0	0	0	0	0	0	0	1	0	0	0	1
-	Meaningful Change	0	0	0	0	1	0	0	0	0	0	0	1
-	Meaningful Difference	0	0	1	0	0	0	0	0	0	0	0	1
-	Meaningful or Important Difference	0	0	0	0	0	1	0	0	0	0	0	1
-	Minimal Important Clinical Change	0	0	0	0	0	0	0	0	0	0	1	1
-	Minimal Meaningful Change Threshold	1	0	0	0	0	0	0	0	0	0	0	1
-	Minimally Clinically Meaningful Change	1	0	0	0	0	0	0	0	0	0	0	1
-	Patient Satisfaction	0	0	0	1	0	0	0	0	0	0	0	1

*Alternative spellings combined: “difference” or “differences”; “minimum”, “minimal” or “minimally”

In addition, the threshold derivation articles were divergent in how thresholds were defined, derived, applied, and interpreted. With respect to terminology, some papers used terms interchangeably, and definitions were often inconsistent or in some cases not provided at all. The definition of the most used term, MCID, varied across papers, with over 50 different references cited for the definition. While most definitions identified the magnitude as “small”, “minimal” or “lowest”, this was not always the case. Some included the notion of “clinical” relevance (to the clinician or patient), while most defined it based solely on the patient’s perception of what is meaningful. In some cases, the definition specified the direction of the change (e.g., “improvement”). Of note, many papers did not specify the intended threshold use (e.g., individual-/group-level) or provide sufficient details on methods for deriving the thresholds, making it impossible to know how the threshold should be applied.

## Discussion

In this article, we aimed to provide the history and evolution of terms used for COA score interpretation thresholds from their origins in the mid to late 1980s all the way through to current practice of use across stakeholders, including individual researchers presenting threshold estimates in COA threshold derivation papers. One of our main objectives was an exploration of current trends that can serve as a basis for harmonizing and streamlining terminology in the field and developing recommendations for the future application of COA score interpretation thresholds.

Our findings clearly highlight the challenge of aligning on terminology for COA score interpretation thresholds given the variability in the literature and inconsistent use by various stakeholders globally. At first glance, differences in terminology may seem like a minor issue. However, our review revealed that inconsistent terminology has led to significant confusion about the meaning of interpretation thresholds and their application, leading to potentially erroneous interpretation of COA results. While we strongly believe that consistent use of terminology will help the wider COA community, we feel that our work cannot result in a clear recommendation of what terms to use without risking adding yet another piece to the current confusion. Rather, our present review should be used as foundation to harmonize and streamline the field. For successful harmonization, efforts must be supported by representation of all relevant COA stakeholders across different fields, specialties, and disease areas, because our review suggests that different stakeholder groups currently use different terms, or even the same terms with different definitions. Therefore, without a wide collaborative effort it would be impossible to persuade all groups to adopt new language. As an interim solution, we recommend that whatever term is used to describe a threshold be clearly defined, so that it can be applied appropriately and replicated. Based on the results of this review, we suggest the following minimum reporting standards be followed to comprehensively define a COA score interpretation threshold (Table 4).

**Table 4:** Minimum reporting standards for defining coa score interpretation thresholds

Characteristic	Recommendation
Perspective	Identify the perspective for what is considered ‘meaningful’ • Patient • Clinician • Observer (e.g., parent, carer)
Application/Use	Identify how the interpretation threshold should be applied • Individual-level or group-level (within-person, within-group, between-group) • Cross-sectionally, longitudinally or both (i.e., cross-sectional between-group difference, change over time within an individual or within a group) • Improvement, worsening or both
Magnitude	Define the magnitude that the interpretation threshold is intended to represent • Minimum value above which a difference/change would be statistically meaningful (i.e., larger than measurement error) • Minimum value above which a difference/change would be considered ‘meaningful’ • Value of ‘substantial’ difference/change (i.e., larger than minimal) • Value beyond which persons consider themselves ‘well’, i.e., “Feeling good rather than feeling better (…)” [93]
Methodology to derive the threshold	Report the approach, method and context used to derive the interpretation threshold • *Approach*, i.e., qualitative, anchor-based (including information on anchors used), distribution- based or a combination • *Method*, e.g., mean change, receiver operating curve (ROC), linear regression, ½ standard deviation (SD) • *Context*, i.e., population, treatment, study design (e.g., observational, randomized controlled)

A strength of this work was the broad scope and large volume of literature reviewed, including methodologic review articles, key stakeholder guidance documents, and original threshold derivation papers. While the review of methodology review papers was updated in September 2024, the review of original threshold derivation papers used the original search. Given the exponential increase in original threshold derivation articles published, the additional volume of literature was too large to be able to update the search. However, the most recent review articles provided similar commentary around the use or misuse of terms, suggesting that the results or conclusions drawn would not have been substantially different. While the scope included different stakeholder perspectives, some perspectives were not included in the review, such as those of professional learned societies and value-based healthcare. As in every concerted effort like this, it is impossible to obtain a representative perspective of every stakeholder group; however, we feel that our inclusion of the various data sources served the purpose of this review which, to our knowledge, is the first of its kind to provide such a comprehensive coverage of different stakeholder perspectives on the given topic. The aim of this article was to provide an overview of the current use of score interpretation thresholds and point to the continued confusion in the field of how to define, use, derive and interpret these thresholds. As described above, an ideal next step would be to gather representatives of each relevant stakeholder group from across disciplines and geographical regions to refine recommendations to get one step closer to consistent terminology and definitions for score interpretation thresholds.

## Conclusions

Our comprehensive review clearly highlighted the vast inconsistency in terms, acronyms, definitions, methods, applications, and interpretations of COA score interpretation thresholds across stakeholders and across therapeutic areas. While this finding was anticipated, the variation across review articles, across the different stakeholder groups and across over 400 interpretation threshold derivation papers was startling. We have provided a recommendation of minimum reporting standards to ensure interpretation thresholds reported in the literature are, at the very minimum, comprehensible and reproducible, regardless of actual terms and acronyms used. However, our work can only be one step of a concerted effort to harmonize and streamline the field. In light of the exponential increase in interest in COA score interpretation thresholds in research and practice, there is a pressing need for all key COA stakeholders from regulatory, industry, clinical practice, academia, as well as patient representatives, to join forces to stipulate definitions and methods to harmonize the field of COA score interpretation thresholds once and for all.

## Data Availability

The data used during the current study are available from the corresponding author upon reasonable request.
